# Supplementary material for: An unbiased, automated platform for scoring dopaminergic neurodegeneration in C. elegans
Source: PLoS One. 2023 Jul 7;18(7):e0281797. doi: 10.1371/journal.pone.0281797 (PMC10328331; doi:10.1371/journal.pone.0281797)
Supplement: S1 Text — (DOCX) [file pone.0281797.s001.docx]

**Analysis of lipid content:** Males from strains LIU1 and LIU2 were obtained by heat-shock and crossed with hermaphrodites from strains RB1600 and RB2237 respectively. Animals were synchronized via egg lays in which adults were transferred to K-agar plates seeded with OP50 E. coli for 3 hours to lay eggs. All non-egg animals were washed from the plate. Animals were allowed to mature for 8 days with daily transfers to fresh plates to ensure separation from progeny. On day 8 worms were imaged via fluorescent microscopy with a Keyence BZ-X710 fluorescence microscope using a 10X objective. Quantification of fluorescence was performed in ImageJ by using the brightfield image to set the entire body of the worm as the region of interest and subsequently measuring mean grey value at the relevant fluorescence wavelength. A section of the image with no worm was also quantified and used for background subtraction.
