# Supplementary material for: An unbiased, automated platform for scoring dopaminergic neurodegeneration in C. elegans
Source: PLoS One. 2023 Jul 7;18(7):e0281797. doi: 10.1371/journal.pone.0281797 (PMC10328331; doi:10.1371/journal.pone.0281797)
Supplement: S1 Table — Size measurements are converted by multiplying pixel counts by the user-provided pixel size (μm/pixel). All feature morphology metrics (area-perimeter) can be more thoroughly explained by reading the MathWorks region props documentation (https://www.mathworks.com/help/images/ref/regionprops.html). (DOCX) [file pone.0281797.s002.docx]

**S1 Table.** Neurodegeneration metrics detected by AUDDIT. Size measurements are converted by multiplying pixel counts by the user-provided pixel size (µm/pixel). All feature morphology metrics (area-perimeter) can be more thoroughly explained by reading the MathWorks region props documentation (https://www.mathworks.com/help/images/ref/regionprops.html).

| Metric | Description | Unit |
| --- | --- | --- |
| Dendrite Remaining | Percentage of dendrite detected by AUDDIT. Measured by dividing the number of pixel rows with detected dendrite by the number of rows between the start and end of the dendrite. | % |
| Dendrite Intensity | Mean pixel value of detected dendrites from masked dendrite image. This value does not include pixel values of breaks. | au |
| Dendrite Length | Number of pixel rows between first and last rows with detected dendrites. Converted to microns with user-provided pixel size. | µm |
| Dendrite Width | Mean width of each row of detected dendrite. | µm |
| Feature Count | Number of detected features on each dendrite. | au |
| Feature Count per Length | Feature count normalized by total dendrite length. | au |
| Weighted Feature Count | Feature count divided by percent dendrite remaining. | au |
| Area | Number of pixels in detected feature count. | µm^2^ |
| Width | Number of pixels across the minor axis length. | µm |
| Intensity | Mean pixel value of detected feature. | au |
| Location | Normalized distance of feature from the cell body of the CEP. | au |
| Eccentricity | Eccentricity of the detected feature ellipse ranging from 0 to 1. 0 is a perfect circle and 1 is a line segment. | au |
| Circularity | Roundness of the detected feature computed as (4*area*pi)/(Perimeter^2^) | au. |
| Orientation | Angle between the x axis of the image and the major axis of the detected feature. | degree |
| Extent | Ratio of pixels in detected feature to the area of pixels of the detected feature’s bounding box. | au |
| Convex Area | Area of the smallest convex polygon surrounding the detected feature. | µm^2^ |
| Solidity | Proportion of pixels in the convex area that are detected as part of the feature. | au |
| Min. Feret Diameter | Minimum distance between any two boundary points. Also known as the caliper diameter. | µm |
| Perimeter | Number of pixels on the border of the detected feature. | µm |
| Break Count | Number of breaks, regardless of length, on the dendrite | au |
