# Supplementary material for: An unbiased, automated platform for scoring dopaminergic neurodegeneration in C. elegans
Source: PLoS One. 2023 Jul 7;18(7):e0281797. doi: 10.1371/journal.pone.0281797 (PMC10328331; doi:10.1371/journal.pone.0281797)
Supplement: S2 Table — (DOCX) [file pone.0281797.s003.docx]

**S2 Table.** p-values for comparisons across groups in 6-OHDA experiments (refer to Figures 5 and S6)

| Dendrite Remaining | | | Feature Intensity | | | Weighted Feature Count | | |
| --- | --- | --- | --- | --- | --- | --- | --- | --- |
| Group 1 | **Group 2** | **Sig** | **Group 1** | **Group 2** | **Sig** | **Group 1** | **Group 2** | **Sig** |
| BY200 Control | *tub-1 Control* | N.S | BY200 Control | *tub-1* Control | N.S | BY200 Control | *tub-1* Control | N.S. |
| BY200 Control | *tub-2* Control | N.S | BY200 Control | *tub-2 Control* | N.S | BY200 Control | *tub-2 Control* | N.S. |
| *tub-1 Control* | *tub-2* Control | N.S | *tub-1* Control | *tub-2* Control | N.S | *tub-1* Control | *tub-2* Control | N.S. |
| BY200 10mM | *tub-1* 10mM | N.S | *BY200 10mM* | *tub-1* 10mM | N.S | BY200 10mM | *tub-1 10mM* | N.S. |
| BY200 10mM | tub-2 10mM | *** | *BY200 10mM* | *tub-2* 10mM | ** | BY200 10mM | *tub-2* 10mM | N.S. |
| *tub-1* 10mM | tub-2 10mM | N.S | *tub-1* 10mM | *tub-2* 10mM | *** | *tub-1 10mM* | *tub-2* 10mM | N.S. |
| BY200 25mM | *tub-1 25mM* | N.S | BY200 25mM | *tub-1* 25mM | N.S | BY200 25mM | *tub-1* 25mM | N.S. |
| BY200 25mM | *tub-2 25mM* | N.S | BY200 25mM | *tub-2* 25mM | N.S | BY200 25mM | *tub-2* 25mM | N.S. |
| *tub-1 25mM* | *tub-2 25mM* | N.S | *tub-1* 25mM | *tub-2* 25mM | N.S | *tub-1* 25mM | *tub-2* 25mM | N.S. |
| *tub-1 25mM* | BY200 50mM | ** | *tub-1* 25mM | BY200 50mM | N.S | *tub-1* 25mM | BY200 50mM | N.S. |
| BY200 50mM | *tub-1* 50mM | N.S | BY200 50mM | *tub-1* 50mM | N.S | BY200 50mM | *tub-1* 50mM | N.S. |
| BY200 50mM | *tub-2* 50mM | * | BY200 50mM | *tub-2* 50mM | N.S | BY200 50mM | *tub-2* 50mM | N.S. |
| *tub-1* 50mM | *tub-2* 50mM | N.S | *tub-1* 50mM | *tub-2* 50mM | *** | *tub-1* 50mM | *tub-2* 50mM | N.S. |
