# Supplementary material for: An unbiased, automated platform for scoring dopaminergic neurodegeneration in C. elegans
Source: PLoS One. 2023 Jul 7;18(7):e0281797. doi: 10.1371/journal.pone.0281797 (PMC10328331; doi:10.1371/journal.pone.0281797)
Supplement: S3 Table — (DOCX) [file pone.0281797.s004.docx]

**S3 Table.** Sample sizes for comparisons across groups in 6-OHDA experiments (refer to Figures 5 and S6)

| Strain | 6-OHDA Exposure | Analysis  Type | N |
| --- | --- | --- | --- |
| BY200 | 0 mM | Manual | 56 |
|  |  | Code | 52 |
|  | 10 mM | Manual | 70 |
|  |  | Code | 60 |
|  | 25 mM | Manual | 68 |
|  |  | Code | 48 |
|  | 50 mM | Manual | 46 |
|  |  | Code | 32 |
| *tub-1* | 0 mM | Manual | 64 |
|  |  | Code | 56 |
|  | 10 mM | Manual | 76 |
|  |  | Code | 68 |
|  | 25 mM | Manual | 67 |
|  |  | Code | 60 |
|  | 50 mM | Manual | 78 |
|  |  | Code | 76 |
| *tub-2* | 0 mM | Manual | 86 |
|  |  | Code | 76 |
|  | 10 mM | Manual | 70 |
|  |  | Code | 56 |
|  | 25 mM | Manual | 78 |
|  |  | Code | 64 |
|  | 50 mM | Manual | 91 |
|  |  | Code | 30 |
