# Supplementary figures and images for: An unbiased, automated platform for scoring dopaminergic neurodegeneration in C. elegans
Source: PLoS One. 2023 Jul 7;18(7):e0281797. doi: 10.1371/journal.pone.0281797 (PMC10328331; doi:10.1371/journal.pone.0281797)

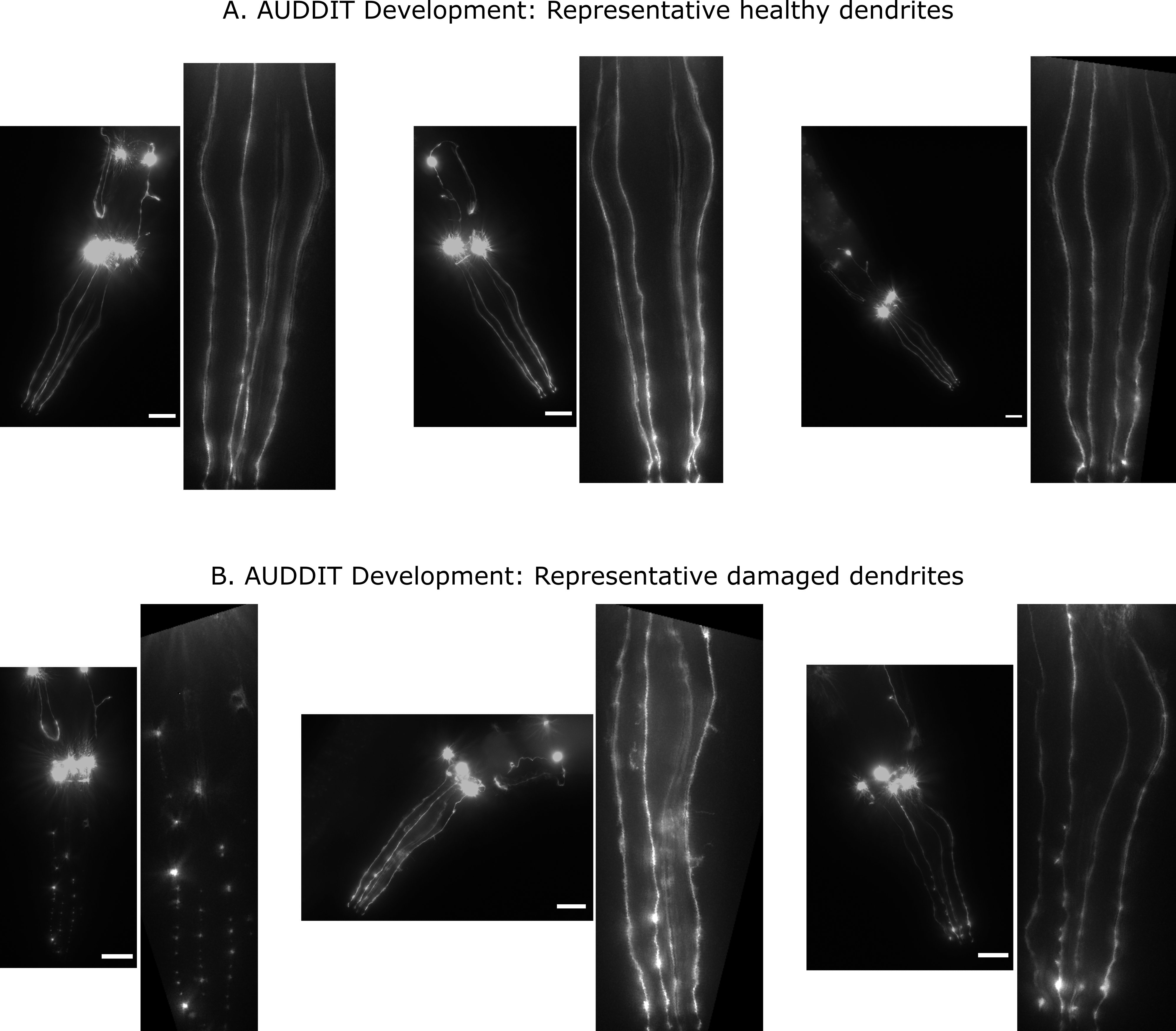

Supplement: S1 Fig — A) Control animals. B) 6-OHDA- exposed CEP dendrites. (PNG) [file pone.0281797.s005.png]

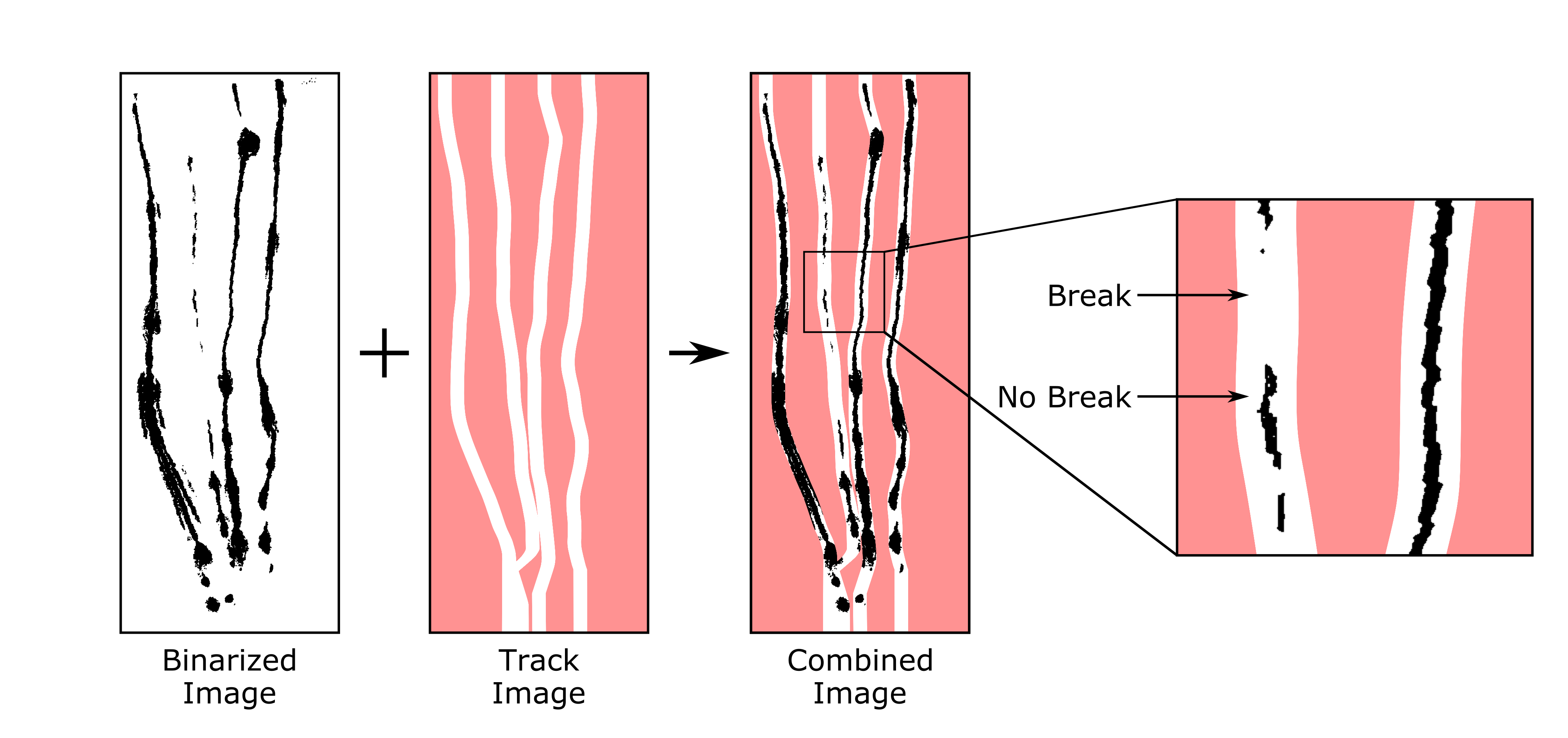

Supplement: S2 Fig — A binarized image of the dendrites is combined with the tracks for each dendrite. Starting from the top of the image, each row in the tracks is analyzed to see if the dendrite is binarized at that row. (PNG) [file pone.0281797.s006.png]

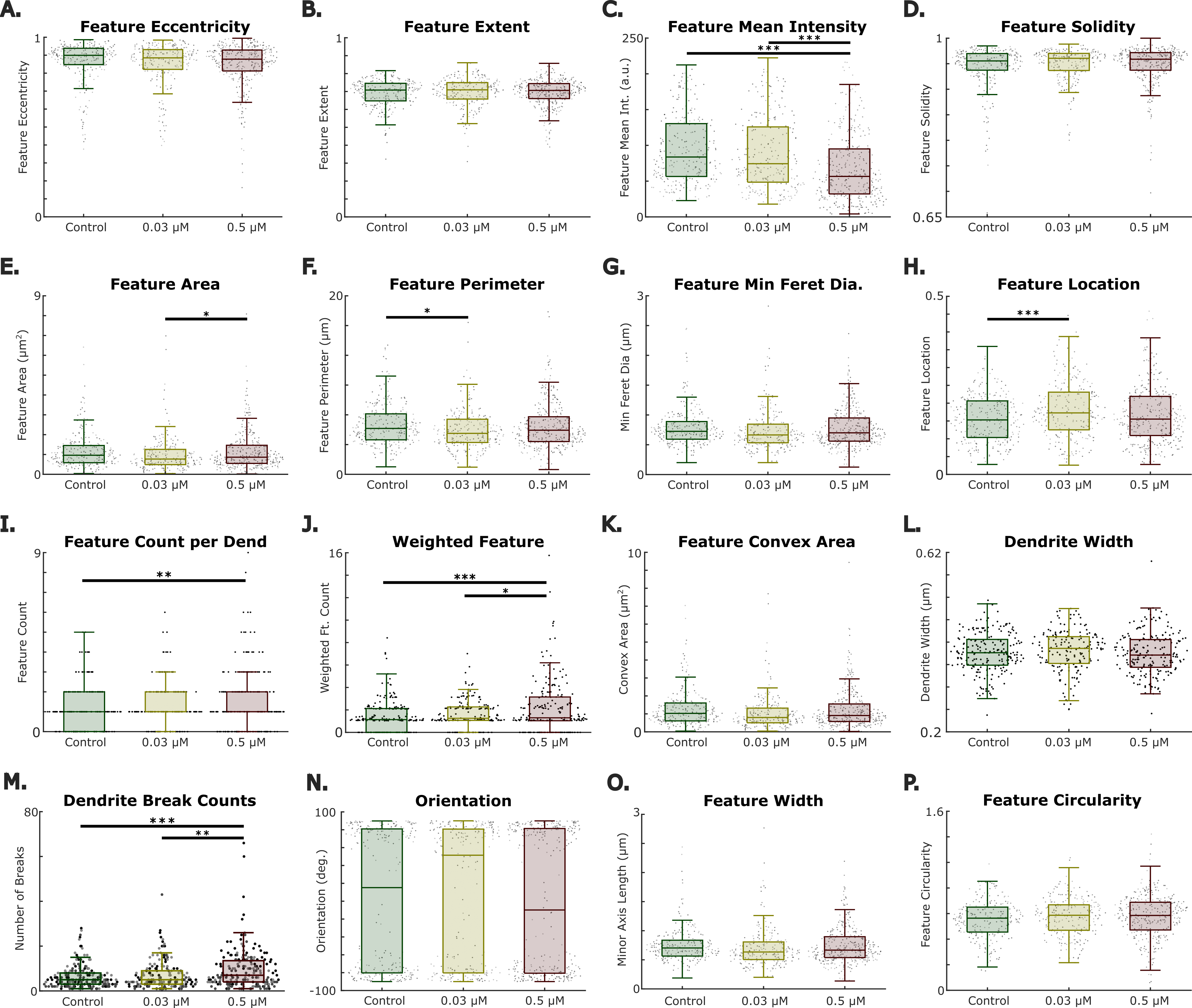

Supplement: S3 Fig — (PNG) [file pone.0281797.s007.png]

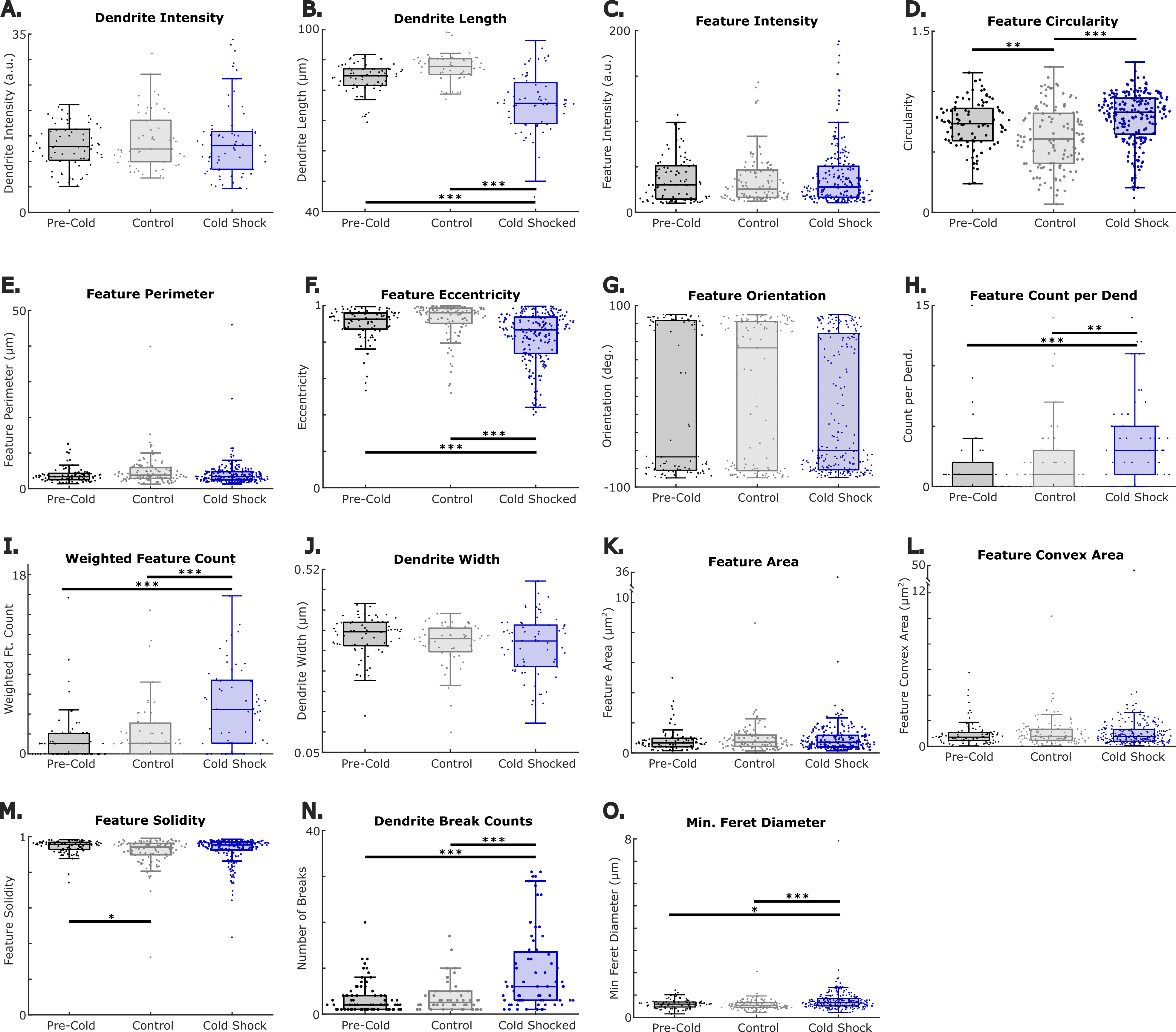

Supplement: S4 Fig — (PNG) [file pone.0281797.s008.png]

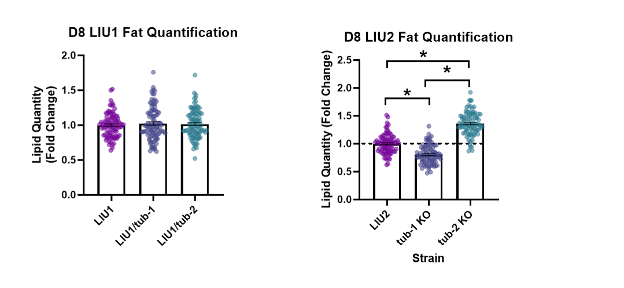

Supplement: S5 Fig — Worms from strain LIU1 (ldrIs1 [dhs-3p::dhs-3::GFP + unc-76(+)]) or LIU2 (ldrIs2 [mdt-28p::mdt-28::mCherry + unc-76(+)]) were independently crossed with tub-1 or tub-2 KO strains to provide two different methods for lipid quantification. LIU1 crosses, in which a GFP fusion protein localizes to lipid droplets in the intestine, shows no difference in fluorescence across controls or mutants (One-way ANOVA with Tukey’s post-hoc, n = 90, 93, 93 respectively. LIU2 crosses, in which the GFP fusion protein localizes to lipid droplets in the intestine, hypodermis, and muscle demonstrate decrease when comparing tub-1 mutants to controls and an increase when comparing tub-2 mutants to controls (One-way ANOVA with Tukey’s Post-hoc, n = 82, 81, 86 respectively). (PNG) [file pone.0281797.s009.png]

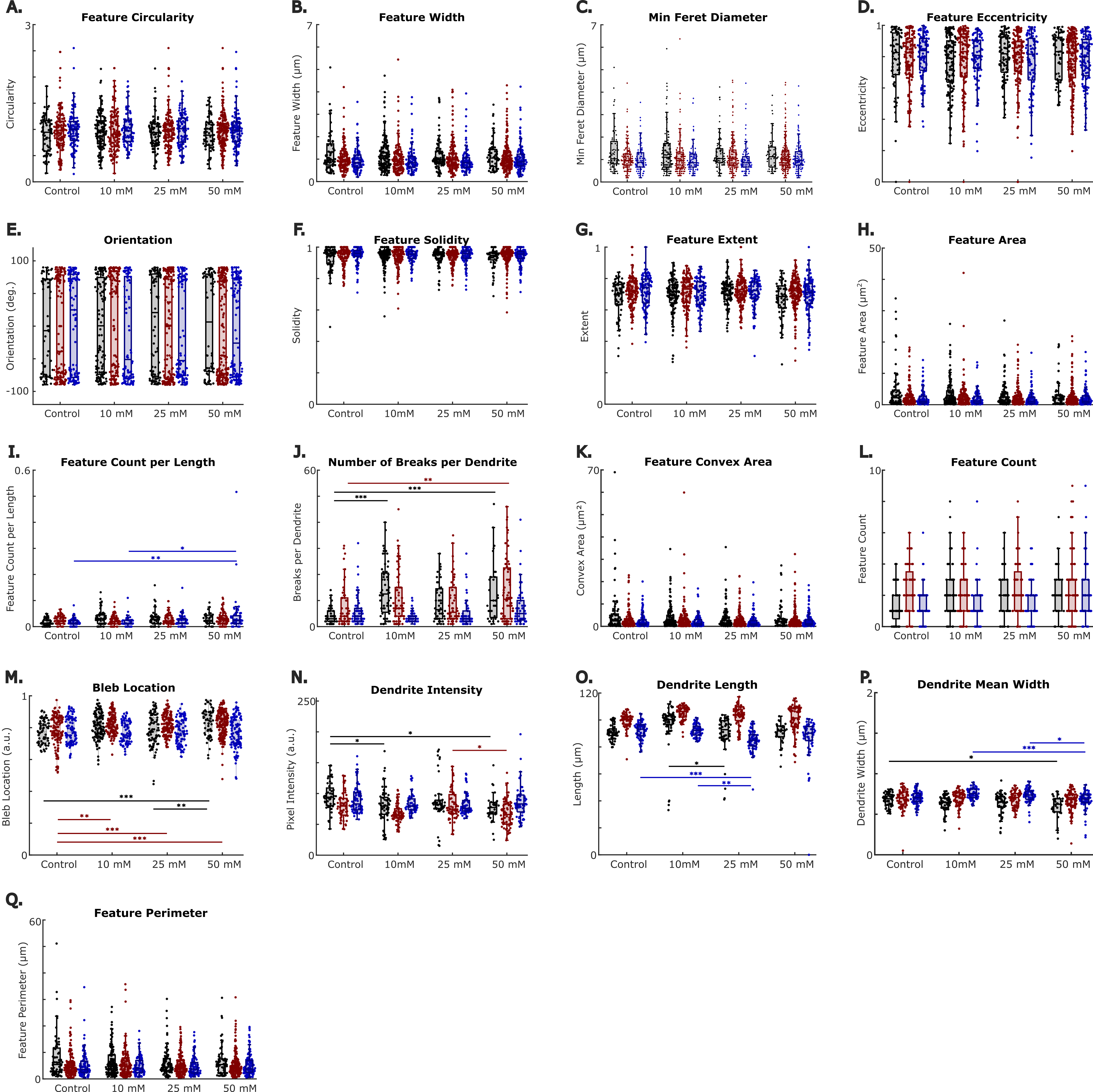

Supplement: S6 Fig — (One-way ANOVA. *p < 0.05; **p < 0.01; ***p < 0.001, additional statistical information provided the Dryad repository). (PNG) [file pone.0281797.s010.png]

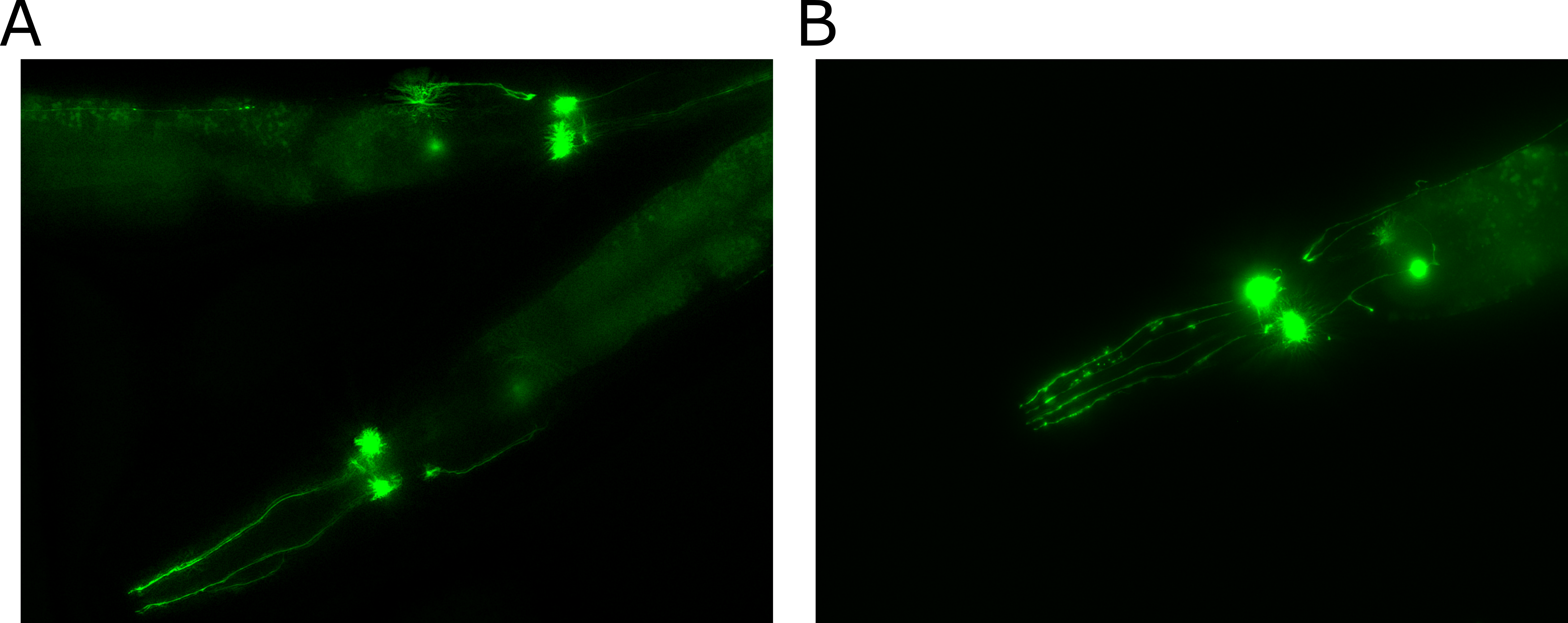

Supplement: S7 Fig — A. An example of an image that would not be accurately analyzed by AUDDIT. The frame of the image contains multiple worms. In addition, the head region of one of the worms is not fully in the frame. The head region of the other worm is oriented such that two of the four CEP dendrites are overlapping, making it impossible to analyze each of them. B. AUDDIT would be able to accurately analyze the CEP dendrites of this worm. The head region of the worm is centered in the image, and there are no other worms or objects around the edges of the frame. The worm is oriented such that all four CEP dendrites are visible, and thus can each be analyzed. (PNG) [file pone.0281797.s011.png]
